# Efficient Isolation of Bacterial RNAs Using Silica-Based Materials Modified with Ionic Liquids

**DOI:** 10.3390/life11101090

**Published:** 2021-10-15

**Authors:** Patrícia Pereira, Augusto Q. Pedro, Márcia C. Neves, João C. Martins, Inês Rodrigues, Mara G. Freire, Fani Sousa

**Affiliations:** 1 CEMMPRE, Department of Chemical Engineering, University of Coimbra, Rua Sílvio Lima-Pólo II, 3030-790 Coimbra, Portugal; papereira@ipn.pt; 2 IPN, Instituto Pedro Nunes, Associação para a Inovação e Desenvolvimento em Ciência e Tecnologia, Rua Pedro Nunes, 3030-199 Coimbra, Portugal; 3 CICECO—Aveiro Institute of Materials, Department of Chemistry, University of Aveiro, 3810-193 Aveiro, Portugal; apedro@ua.pt (A.Q.P.); mcneves@ua.pt (M.C.N.); 4 CICS-UBI—Health Sciences Research Centre, Universidade da Beira Interior, 6200-506 Covilhã, Portugal; joao_martins1994@hotmail.com (J.C.M.); ines_rodrigues@hotmail.com (I.R.)

**Keywords:** adsorption kinetics, downstream processes, liquid chromatography, RNA, silica, supported ionic liquids

## Abstract

High quality nucleic acids (with high integrity, purity, and biological activity) have become indispensable products of modern society, both in molecular diagnosis and to be used as biopharmaceuticals. As the current methods available for the extraction and purification of nucleic acids are laborious, time-consuming, and usually rely on the use of hazardous chemicals, there is an unmet need towards the development of more sustainable and cost-effective technologies for nucleic acids purification. Accordingly, this study addresses the preparation and evaluation of silica-based materials chemically modified with chloride-based ionic liquids (supported ionic liquids, SILs) as potential materials to effectively isolate RNAs. The investigated chloride-based SILs comprise the following cations: 1-methyl-3-propylimidazolium, triethylpropylammonium, dimethylbutylpropylammonium, and trioctylpropylammonium. All SILs were synthesized by us and characterized by solid-state ^13^C Nuclear Magnetic Resonance (NMR), Scanning Electron Microscopy (SEM), elemental analysis, and zeta potential measurements, confirming the successful covalent attachment of each IL cation with no relevant changes in the morphology of materials. Their innovative application as chromatographic supports for the isolation of recombinant RNA was then evaluated. Adsorption kinetics of transfer RNA (tRNA) on the modified silica-based materials were investigated at 25 °C. Irrespective to the immobilized IL, the adsorption experimental data are better described by a pseudo first-order model, and maximum tRNA binding capacities of circa 16 µmol of tRNA/g of material were achieved with silica modified with 1-methyl-3-propylimidazolium chloride and dimethylbutylpropylammonium chloride. Furthermore, the multimodal character displayed by SILs was explored towards the purification of tRNA from *Escherichia coli* lysates, which in addition to tRNA contain ribosomal RNA and genomic DNA. The best performance on the tRNA isolation was achieved with SILs comprising 1-methyl-3-propylimidazolium chloride and dimethylbutylpropylammonium chloride. Overall, the IL modified silica-based materials represent a more efficient, sustainable, and cost-effective technology for the purification of bacterial RNAs, paving the way for their use in the purification of distinct biomolecules or nucleic acids from other sources.

## 1. Introduction

Biopharmaceuticals make up about one-third of drugs currently in development and refer to pharmaceutical substances derived from biological sources with clinical efficacy. Among these, nucleic-acids-based biopharmaceuticals are a fast-growing area with significant relevance [1,2].

Over the past 20 years, we have witnessed remarkable discoveries in the biological functions of RNA, which is being increasingly perceived as a major regulator of a plethora of biological processes. Currently, RNA is regarded as an important drug target and the RNA-based biopharmaceuticals market is expected to assume more relevance in the future, a tendency that seems to start being confirmed due to the worldwide distribution of messenger RNA for tackling the COVID-19 pandemics [3].

In parallel with developments in biotechnology and clinical studies, the importance of obtaining pure RNA samples has greatly increased once it is a critical step affecting many techniques such as reverse transcriptase polymerase chain reaction, cDNA library construction, Northern blot, and microarrays analysis and also their application as biopharmaceuticals [4,5]. However, to make biopharmaceuticals (including those based on RNA molecules) accessible to a wider population, it is essential to decrease their manufacturing costs, which can be achieved with the establishment of cost-effective purification platforms. In the past decade, significant advances in biopharmaceuticals purification technologies allowed the development of novel and improved strategies [6]; however, considering the strict quality criteria (identity—should be confirmed after manufacturing; purity—testing for bacterial endotoxins and process/product associated impurities; safety —tests for endotoxins, bacterial and fungal sterility or bioburden; stability—to establish shelf-life and appropriate storage conditions; potency—in vitro, in vivo tests, or both and based on individual product attributes) that must be fulfilled envisioning therapeutic applications [7,8], there is an unmet need to surpass technical challenges while envisaging products that fulfill the guidelines of regulatory agencies. The main drawbacks are usually related to the capacity of handling the increasing concentration of products in the crude feedstock, the ability to reach a high purity degree, and the possibility to integrate primary isolation and purification steps, while guaranteeing that the structure and biological activity of these bioproducts are preserved [9,10].

In the last few years, novel bioseparation methods for the purification of nucleic acids have been proposed [10,11,12]. Among these, affinity chromatography-based strategies have been addressed by exploring the interactions occurring between nucleic acids (pDNA and RNA) and amino acids (or its derivatives), which act as chromatographic ligands immobilized onto distinct types of materials (agarose beads, monoliths, and macroporous matrices) [13]. However, the selectivity, specificity, capacity, and robustness of these ligands are not as high as desirable, reinforcing the need to develop novel strategies comprising ligands with improved performance as is the case of ionic liquids (ILs).

ILs were initially proposed as more environmentally friendly alternatives to conventional volatile organic solvents due to their non-flammability and negligible volatility at ambient conditions. ILs are organic salts composed of organic cations and organic or inorganic anions, thus displaying low melting temperatures. ILs, if properly designed, further display high thermal and chemical stabilities, and are usually referred as tunable designer solvents, in which, by combining different cation–anion pairs, their characteristics can be tailored to meet the requirements of a given application [14,15,16,17,18]. These solvents, which can be liquid at room temperature, have been applied in distinct fields, ranging from (bio)catalysis to extraction and separation techniques [14,15,16,17,18]. Furthermore, a lot of evidence has shown that ILs maintain the structure and integrity of biologically active biomolecules, such as DNA [19,20], proteins [21,22], and RNA [23,24,25]. In this field, our research group previously reported pioneering results on the enhanced stability of bacterial transfer RNAs afforded by aqueous solutions of ILs [24,25]. On the other hand, by taking advantage of the chemical diversity shown by ILs, a variety of materials (like silica, carbon nanotubes, polymers) have been chemically modified with ILs, resulting in the known supported ionic liquids (SILs). These materials retain the designer solvent character displayed by ILs and have been successfully applied in the separation of anions, aromatic carboxylic acids, amines, and nucleotides [26,27,28]. More recently, we have reported the functionalization of a macroporous (non-silica-based) chromatographic support with the IL 1-methyl-3-propylimidazolium chloride that displays a remarkable performance to purify nucleic acids [29]. Herein, we report the chemical modification of silica-based stationary phases with different ILs, namely with 1-methyl-3-propylimidazolium chloride, triethylpropylammonium chloride, dimethylbutylpropylammonium chloride, and trioctylpropylammonium chloride. Although the synthesis process using amorphous silica was previously reported [28,29,30], their application as chromatographic matrices for the separation of nucleic acids is addressed here for the first time. The scope of SILs towards the separation of nucleic acids is extended here, both by the preparation of SILs with distinct chemical structures and by the study of distinct chromatographic conditions, allowing for inferring the role that the IL chemical structure plays in the nucleic acids interaction and purification.

## 2. Materials and Methods

### 2.1. Chemicals and Reagents

All chemicals and reagents were purchased in the highest purity commercially available and used as received unless otherwise noted. The reagents used in the preparation of IL-functionalized silica were silica gel (60, 0.2–0.5 mm) acquired from Merck (Darmstadt, Germany); hydrochloric acid (37% *w*/*w)* and *N,N*-dimethylbutylamine (99%) obtained from Sigma-Aldrich (St. Louis, MO, USA); toluene (99.8%) and triethylamine (HPLC grade) purchased from Fisher Chemical (Waltham, MA, USA); ethanol (99.9%) from Carlo Erba (Milan, Italy); methanol (HPLC grade) from Chem-Lab (Zedelgem, Belgium); trioctylamine (98%) from Fluka (Cambridge, UK); and (3-chloropropyl)trimethoxysilane (98%) and N-methylimidazole (99%) from Acros Organics (Geel, Belgium). For the bacterial growth of the strain *E. coli* DH5α, yeast extract and tryptone were purchased from Bioakar (Beauvais, France), Himedia’s glycerol and potassium hydrogen phosphate (K_2_HPO_4_) from Panreac (Barcelona, Spain), potassium dihydrogen phosphate (KH_2_PO_4_) from Sigma-Aldrich (St. Louis, MO, USA) and Luria-Broth Agar medium from Pronalab (Merida, Mexico). In sample processing, all chemicals used in the cell lysis buffer were from Sigma-Aldrich, while β-mercaptoethanol was obtained from Merck (Whitehouse Station, NJ, USA).

The reagents used in chromatographic experiments were ammonium sulfate and sodium chloride, purchased from Panreac (Barcelona, Spain), and tris(hydroxymethyl)aminomethane (Tris) from Merck (Darmstadt, Germany). All buffers used for the chromatographic experiments were freshly prepared with ultra-pure grade deionized water purified in a Milli-Q system from Millipore (Billerica, MA, USA) treated with 0.05% diethylpyrocarbonate (DEPC) from Sigma-Aldrich (St. Louis, MO, USA). Buffers were filtered through a 0.20-μm pore size membrane (Whatman, Dassel, Germany) and degassed ultrasonically before use. HyperLadder I (Bioline, London, UK) was used as DNA molecular weight marker. GreenSafe Premium stain for nucleic acid gel electrophoresis was purchased from NZYTech (Lisbon, Portugal).

### 2.2. Chemical Modification of Silica with Ionic Liquids

The preparation of the chromatographic supports was achieved by the chemical modification of silica with each IL and in accordance with procedures previously reported by our research team [28,29,30]. Briefly, 5 g of silica were initially immersed in hydrochloric acid (37%) for 24 h, and then washed with double distilled water and dried under vacuum at 105 °C to ensure its activation. The second stage of the reaction involves the dispersion of the activated silica in 60 mL of toluene, followed by the addition of 5 mL of 3-chloropropyltrimethoxysilane. The reaction solution was magnetically stirred (550 rpm) and heated (115 °C) under reflux for 24 h. After refluxing, the reaction was stopped and the modified silica was cooled down to room temperature and washed with 100 mL of toluene, 200 mL of ethanol-water (1:1, *v*/*v*) mixture, 500 mL of distilled water, and 100 mL of methanol. Then, the material was dried under vacuum at 60 °C for 24 h. Aiming the chemical modification of silica with 1-methyl-3-propylimidazolium chloride, the dried chloropropyl silica ([Si][C_3_]Cl) was then mixed with 5 mL of 1-methylimidazol and 60 mL of toluene, and the reaction proceeded for 24 h under reflux at 107 °C with 550 rpm of stirring.

**Figure 1:**
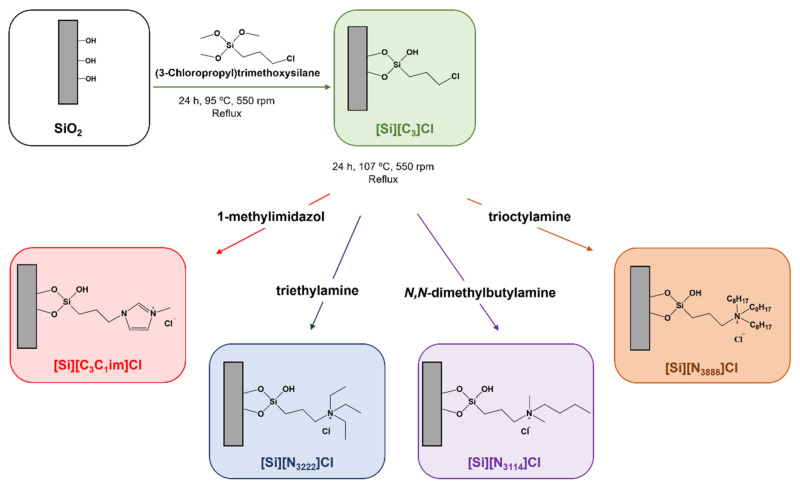
Schematic representation of the preparation of IL-functionalized silica supports, i.e., SILs.

To achieve the preparation of SILs, triethylamine, *N,N*-dimethylbutylamine and trioctylamine were, respectively, reacted with the chloropropyl silica and treated as previously described. The reaction was stopped, and the modified silica was cooled down to room temperature, and washed with 100 mL of methanol, 200 mL of ethanol–water (1:1, *v*/*v*) mixture, 500 mL of double distilled water, and 100 mL of methanol. Each functionalized silica was dried under vacuum at 60 °C for 24 h prior to their characterization and further use. A schematic diagram of the synthetic approach used for the preparation of IL-functionalized silica is shown in Figure 1. Figure 1 also describes the abbreviation of each SIL according to the covalently attached IL cation.

### 2.3. Characterization of SILs

To confirm the correct functionalization of silica with the different ILs, each SIL was characterized by solid-state ^13^C NMR, elemental analysis and zeta potential measurements. SEM was used to appraise possible changes in morphology of the materials.

#### 2.3.1. Solid-State ^13^C NMR Experiments

Solid-state ^13^C NMR analysis of the prepared materials was performed at room temperature using a Bruker Avance III—400 MHz spectrometer (DSX model). A sample of about 100 mg was placed in a ZrO_2_ rotator 4 mm in diameter, which enabled spinning of the sample. The ^13^C CPMAS NMR spectra were recorded at 100.63 MHz in 4 mm BL crosspolarization magic angle spinning (CPMAS) VTN probes. Spectra were processed using Bruker Topspin 3.2.

#### 2.3.2. Elemental Analysis

A 2 mg portion of all samples ([Si]C_3_]Cl, [Si][C_3_C_1_im]Cl, [Si][N_3222_]Cl, [Si][N_3114_]Cl and [Si][N_3888_]Cl) underwent elemental analysis using a TruSpec 630-200-200, a combustion furnace temperature of 1075 °C and afterburner temperature of 850 °C. Carbon, hydrogen, nitrogen and sulfur contents (in weight percentage) were determined using a detection method of infrared absorption for carbon, hydrogen, and nitrogen, and thermal conductivity for nitrogen.

#### 2.3.3. Zeta Potential

The zeta potential of synthesized SILs was determined as a function of pH using suspensions of SILs in water at different pH values. To adjust the pH values, 0.01 M solutions of NaOH and HCl were used, and measurements were carried at 25 °C using a Zetasizer Nano-ZS (Malvern Instruments, Worcestershire, UK). The value of the zeta potential was calculated from the Smoluchowski equation [31].

#### 2.3.4. Scanning Electron Microscopy

Scanning electron microscopy (SEM) was performed using a Hitachi SU70 microscope equipped with EDX Bruker, model Quantax 400. A carbon thin film deposition was used to increase the conductivity of the samples.

### 2.4. Escherichia coli DH5α Culture for Nucleic Acids Production

The nucleic acids samples used in these experiments were obtained from a cell culture of *E. coli* DH5α. The fermentation was carried out in 1 L shake flasks containing 0.25 L of Terrific Broth medium (12 g/L Tryptone, 24 g/L Yeast extract, 4 mL/L glycerol, 0.017 M KH_2_PO_4_ and 0.072 M K_2_HPO_4_) at 37 °C and shaking at 250 rpm.

Cell growth was suspended after 3 h of fermentation in the early exponential phase—Optical density (600 nm) circa 2—to obtain the sample composed of gDNA, rRNA, and tRNA or after 7 h of fermentation in the late exponential phase—Optical density (600 nm) circa 6—to obtain gDNA and tRNA. After the appropriate incubation period, cells were collected by centrifugation at 4500× *g* for 15 min and stored at −20 °C until use.

### 2.5. Isolation of Nucleic Acids from Escherichia coli DH5α

Aiming the isolation of both tRNA and bacterial lysates containing tRNA, rRNA, and gDNA, the corresponding bacterial cells were lysed using the lysis solution reported by Chomczynski and Sacchi [32] with some modifications as previously described [24]. Briefly, cell lysis of a bacterial pellet from 100 mL of culture medium was accomplished by successive pipetting the pellet in 5 mL of denaturing cell lysis solution (4 M guanidinium thiocyanate; 0.025 M sodium citrate, pH 7.0; 0.5% (*w*/*v*) N-laurosylsarcosine and 0.1 M β-mercaptoethanol). For the isolation of tRNA, after an incubation period of 10 min on ice, cellular debris, genomic DNA, and proteins were precipitated by adding 5 mL of water-saturated phenol and 0.5 mL of 2 M sodium acetate (pH 4.0). The isolation of tRNA was achieved by adding 1 mL of chloroform/isoamyl alcohol (49:1, *v*/*v*), and by vigorously mixing until two immiscible phases were obtained. The upper aqueous phase, which contains mostly tRNA, was recovered and concentrated by the addition of 5 mL of ice-cold isopropanol. Precipitated molecules were recovered by centrifugation at 10,000× *g* for 20 min at 4 °C, and resuspended in 1.5 mL of denaturing cell lysis solution. tRNA molecules were concentrated again with 1.5 mL of ice-cold isopropanol. After centrifugation for 10 min at 10,000× *g* (4 °C), the RNA pellet was washed with 7.5 mL of 75% ethanol and incubated at room temperature for 10 min, followed by a 5 min centrifugation at 10,000× *g* (4 °C). The air-dried RNA pellet was solubilized in 1 mL of 0.05% DEPC-treated water.

To obtain the bacterial lysate sample, the suspension containing bacterial cells and denaturing cell lysis solution was incubated on ice for 10 min and centrifuged at 16,000× *g* for 30 min at 4 °C to remove insoluble material. The supernatant containing nucleic acids was collected in a clear lysis tube, mixed with 5 mL of ice-cold isopropanol for nucleic acids precipitation and kept on ice for 30 min. After the centrifugation at 16,000× *g* for 20 min at 4 °C, the resulting pellet was washed with 75% ethanol in DEPC-treated water and incubated at room temperature for 10 min. In the last step, the mixture was centrifuged at 16,000× *g*, 4 °C for 5 min and the nucleic acid pellet was air-dried at room temperature for 15 min. The pellet was used as nucleic acid extract after dissolving in 2 mL of 0.05% DEPC-treated water and incubated for 10 min at 60 °C to ensure complete solubilization. Finally, the final suspension was centrifuged at 16,000× *g* for 30 min at 4 °C, and the supernatant was carefully decanted and properly stored. The absorbance of the samples was determined at 260 and 280 nm using a NanoPhotometer spectrophotometer (IMPLEN, Staffordshire, UK) to assess its concentration. The samples were stored at −80 °C.

### 2.6. Agarose Gel Electrophoresis

The samples obtained after cell lysis and chromatographic assays were analysed by horizontal agarose gel electrophoresis (Hoefer, San Francisco, CA, USA). Briefly, 20 μL of each sample was added to loading dye and injected into individual wells of 0.8% agarose gel in Tris-acetic acid buffer (40 mM Tris base, 20 mM acetic acid and 1 mM EDTA, pH 8.0). The samples run on the gel at 120 V for 30 min. The agarose gel was pre-stained with greensafe (0.5 µg/mL), and the bands were visualized under ultraviolet light used on a Uvitec Cambridge Fire-Reader UV equipped with a camera (UVITEC Cambridge, Cambridge, UK).

### 2.7. RNA Binding Studies

Binding of tRNA to the IL-functionalized silica supports was investigated in a batch mode. An aqueous solution of tRNA at 5 µg/mL was used to determine the RNA binding ability of the prepared supports and experiments were performed as follows: 0.2 g of prepared IL-functionalized silica supports were mixed with 10 mL of RNA aqueous solutions in 50 mL Erlenmeyer, and then placed in an orbital shaker at 120 rpm and 25 °C. The effect of distinct experimental conditions, such as different types of salts (ammonium sulphate and sodium chloride) on tRNA binding, was evaluated. For these studies, different periods of equilibration ranging from 0 to 90 min were investigated. After the binding stage, the IL-support/RNA mixture was centrifuged at 5000 rpm for 5 min, and the amount of tRNA in the supernatant was quantified by measuring the absorbance of all samples at a wavelength of 260 nm through UV-spectroscopy, using a Pharmacia Biotech Ultrospec 3000 UV/Visible Spectrophotometer (Cambridge, UK), where an absorbance (A) of 1 corresponds to a concentration of 40 µg/mL for RNA. At least 3 individual samples for each condition were investigated.

### 2.8. Adsorption Kinetics

Adsorption kinetics of the IL-functionalized silica supports were also investigated in a batch mode, as mentioned above (see Section 2.7). Pseudo-first order (Equation (1)), pseudo-second order (Equation (2)) and Elovich (Equation (3)) reaction-based models were applied to the experimental kinetic data with the purpose of determining the mechanisms controlling tRNA binding process such as mass transfer and chemical reaction [33]. The kinetics models are described by the following equations:

(1) $$q_{t}=q_{e}\left(1-e^{-k_{1}t}\right)$$

(2) $$q_{t}=\frac{q_{e}^{2}k_{2}t}{1+q_{e}k_{2}t}$$

(3) $$q_{t}=\frac{1}{\beta }ln\left(1+\alpha \beta t\right)$$

in which *q_e_* is the experimental amount of tRNA bound at equilibrium (mg/g); *q_t_* is the amount of tRNA bound at time t (mg/g); *k*_1_ (min^−1^) is the pseudo-first order (PFO) rate constant; *k*_2_ (g mg^−1^ min^−1^) the pseudo-second order (PSO) rate constant; *α* (mg g^−1^ min^−1^) the initial sorption rate and *β* (g mg^−1^) the desorption constant.

### 2.9. Binding Experiments of Bacterial Nucleic Acids in the IL-Functionalized Supports in Batch Mode

Binding/elution chromatographic experiments of RNA on the several supports were initially performed in a batch mode and with 0.2 g of each support. Due to the diversity of interactions that ILs can promote with tRNA, several binding/elution strategies were tested. In this way, aiming to promote mainly hydrophobic interactions, the following chromatographic buffers were used: 2 M ammonium sulfate [(NH_4_)_2_SO_4_] in 10 mM Tris-HCl (pH 8) as equilibrium and binding buffer and 10 mM Tris-HCl and 2 M sodium chloride (NaCl) in 10 mM of Tris-HCl as elution buffers. On the other hand, to mainly promote ionic interactions, the following chromatographic buffers were used: 10 mM Tris-HCl as equilibrium and binding buffer and 2 M NaCl in 10 mM of Tris-HCl as elution buffer. In all experiments, 1 mL of equilibrium buffer was added to each silica in study, followed by 10 s of vortex. After centrifugation at 5000× *g* for 2 min, the supernatant was removed and, subsequently, 100 μg/mL of RNA in binding buffer was added. The mixture was incubated for 10 min at 4 °C with slow stirring, to promote the binding of tRNA to the corresponding support. After centrifugation, the supernatant was collected, and 1 mL of elution buffer was added, followed by slow stirring for 10 min at 4 °C.

After each use, supports were washed with DEPC-H_2_O and regenerated by washing consecutively with 0.2 M NaOH and 0.5 M HCl. Posteriorly, the supernatants were collected, concentrated to a volume of 100 μL and desalted using Vivaspin concentrators (Sartorius, Gottingen, Germany). The absorbance of the fractions recovered during the binding and elution steps was measured at 260 nm using a Pharmacia Biotech Ultrospec 3000 UV/Visible Spectrophotometer (Cambridge, England) and analysed by agarose gel electrophoresis. The binding and elution conditions which favor the separation of nucleic acids from a bacterial lysate sample (rRNA, tRNA and gDNA) were optimized using 0.2 g of IL-functionalized silica supports, and testing conditions that promote mainly hydrophobic or electrostatic interactions, as described earlier.

## 3. Results

### 3.1. Characterization of IL-Functionalized Silica Supports

Previously, our research group prepared SILs comprising ILs with distinct chemical structures by the immobilization of the IL cationic moieties in silica for the efficient removal of acetylsalicylic acid from aqueous solutions [30]. In the current work, similar and new SILs were investigated as stationary phases for the separation of nucleic acids, while addressing the type of interactions (assessed using different chromatographic buffers) that are established, as well as their binding capacity towards RNA. Silica was chosen as the stationary phase due to its remarkable properties, namely a high surface area, high thermal and mechanical stabilities, and low-cost [34].

Aiming for the isolation of tRNA from bacterial lysates, silica-based materials functionalized with ILs were prepared by the modification of chloropropyl silica to obtain the following SILs: [Si][C_3_C_1_im]Cl, [Si][N_3222_]Cl, [Si][N_3114_]Cl and [Si][N_3888_]Cl (cf. Figure 1). To confirm the successful immobilization of each IL in the corresponding SIL and to appraise possible changes in morphology of the materials, the functionalized materials were first subjected to chemical and morphological characterizations, namely by solid-state ^13^C RMN, elemental analysis, zeta potential, and SEM, whose results are shown below.

#### 3.1.1. Solid-State ^13^C NMR Analysis

**Figure 2:**
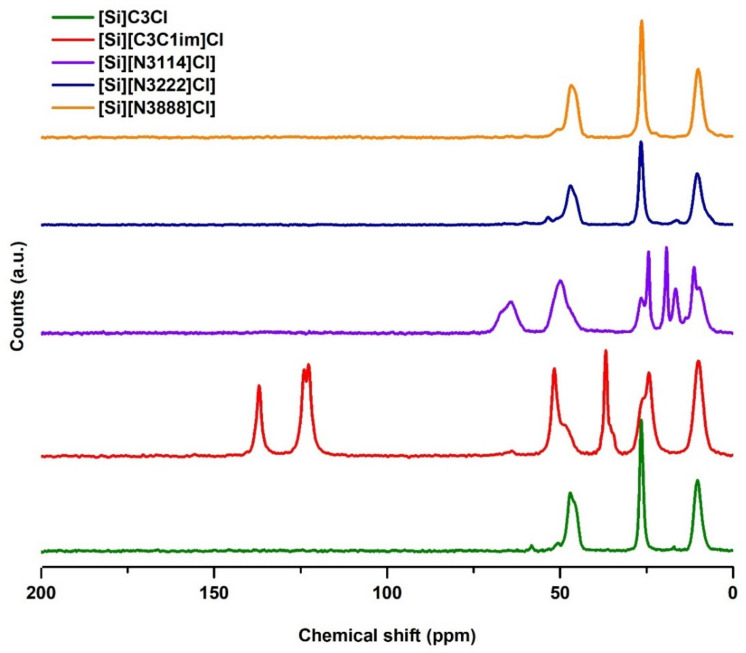
^13^C NMR solid-state spectrum of [Si][C_3_]Cl and IL-functionalized silica supports.

The successful preparation of the SILs was confirmed through solid-state ^13^C NMR, as shown in Figure 2. Unlike IL-functionalized silica, the ^13^C NMR spectrum of the chloropropyl silica does not contain characteristic signals originating from moieties present in the ILs, confirming the effectiveness of the modification process. The NMR spectra for all samples of SILs exhibit three similar signals at 10, 24, and 51 ppm, which are attributed to the three carbon atoms of the propyl alkyl chain of the chloropropyl silica.

In the [Si][C_3_C_1_im]Cl spectrum, there is an additional signal at 37 ppm, which corresponds to the carbon of the methyl chain. The ^13^C NMR spectra for [Si][C_3_C_1_im]Cl also contains characteristic signals at chemical shifts of 120–140 ppm, attributed to the aromatic carbon atoms of imidazolium, proving that the correct functionalization of these supports occurred. The spectrum of the [Si][N_3222_]Cl sample shows the presence of quaternary ammonium methyl groups at chemical shifts of 58 ppm. Regarding the [Si][N_3222_]Cl and [Si][N_3888_]Cl supports, the NMR results alone are not sufficient to prove that the functionalization occurred because, due to their structures, the chemical shifts of the triethylammonium and trioctylammonium cations carbons are similar to the chemical shifts of the propyl alkyl chain carbons from the chloropropyl silica. Still, the following results confirm this IL functionalization.

#### 3.1.2. Elemental Analysis

Elemental analysis was performed to further evaluate if chemical modification of silica with cation sources was properly carried out. To this end, the content of carbon, nitrogen, and hydrogen for the four IL-functionalized silica supports and for the chloropropyl silica ([Si][C_3_]Cl) material were quantitatively determined, the results being provided in Table 1. In general, the elemental analysis of the IL-functionalized silica reveals changes in the relative atomic composition in comparison with that from the chloropropyl silica. The carbon content (wt%) in [Si][C_3_]Cl is 4.73 and no nitrogen was detected, thereby supporting the absence of IL functional moieties in this starting material. On the other hand, for the IL-functionalized silica supports, nitrogen was detected in all modified supports and the content (wt%) of carbon differs from that in the [Si][C_3_]Cl, which confirms the presence of the IL and the effectiveness of the proposed method of immobilization.

According to Table 1, the carbon and nitrogen contents on the IL-functionalized silica supports range from 6.34 to 8.21 wt% and from 0.06 to 2.50 wt%, respectively. These values are in good agreement with those previously reported for some of the SILs addressed in this work [30]. Moreover, based on the nitrogen content provided by the elemental analysis, the ligand density was determined for each modified support [35], being given in Table 1. These values are useful to address the tRNA binding ability and nucleic acids separation performance (addressed below). The highest ligand density was achieved for [Si][C_3_C_1_im]Cl, also in accordance with the higher nitrogen content that this material displays. For the tetraalkylammonium-based materials, the ligand density decreases with the alkyl side chain length increase. Overall, all these results prove that the IL cations were correctly immobilized on the silica surface.

#### 3.1.3. Zeta Potential Analysis

To complete the SIL characterization, changes in zeta potential values as a function of pH were appraised, while determining their point of zero charge (PZC) (see Table 1 and Figure S1 in the Supplementary Material). The functionalized materials present a different behavior as a function of pH in comparison to chloropropyl silica ([Si][C_3_]Cl), indicating that this variation is due to the covalent bonding of the IL cation, causing a charge alteration on the silica surface. [Si][C_3_C_1_im]Cl, [Si][N_3222_]Cl and [Si][N_3114_]Cl matrices have PZC values higher than 9, indicating the correct immobilization of these ILs to the silica, and thus confirming the result obtained by elemental analysis and NMR. As the PZC values are indicative of the superficial charge of the supports, they provide relevant information to select the pH of the chromatographic buffers (addressed below). The PZC value recorded for the [Si][N_3888_]Cl material, complemented with the results already presented for the elemental analysis and NMR, are indicative that a lower number of IL ligands are present per surface area of the material, being in agreement with the ligand density values provided in Table 1.

#### 3.1.4. Scanning Electron Microscopy

To evaluate the morphology of the prepared materials, SEM analysis was performed. SEM images of starting silica and SILs are given in the Supplementary Material (Figure S2). As reported by Bernardo and co-workers [30], no significant differences in the materials morphology were observed between the prepared SILs and the non-functionalized silica. Thus, these findings indicate that the silica functionalization with ILs and required reaction steps do not change the material morphology.

### 3.2. Adsorption Kinetics and Diffusion Models of tRNA in IL-Modified Supports

To evaluate the time required for adsorption and to set the appropriate contact time for tRNA to reach the equilibrium between liquid and solid phases, the adsorption curves of tRNA onto the IL-functionalized silica-based supports were determined. According to the chemical structures of the ILs under study, namely the presence of aromatic cations, the length of alkyl side chains in tetraalkylammonium cations and positive charge centers, the establishment of hydrophobic and electrostatic interactions (among others) with tRNA is expected, which in addition to the negative charge conferred by phosphate groups also contains aromatic moieties due to the presence of nitrogenous bases. Based on the exposed, the effect of different salts and binding/elution strategies on RNA binding onto the modified supports was investigated. Buffers composed of 10 mM Tris-HCl, pH 8.0, and 2 M (NH_4_)_2_SO_4_ in 10 mM Tris-HCl, pH 8.0 were initially chosen to test the tRNA interactions, using an initial tRNA concentration of 5 µg/mL.

Initial results demonstrate that tRNA binding to [Si][C_3_C_1_im]Cl, [Si][N_3222_]Cl and [Si][N_3114_]Cl is achieved with 10 mM Tris-HCl, pH 8.0, as binding buffer. However, [Si][N_3888_]Cl shows an opposite performance and tRNA binding is only achieved using a buffer composed of 2 M (NH_4_)_2_SO_4_ in 10 mM Tris-HCl, pH 8.0 (data not shown). This behavior is in full agreement with the IL chemical structures, in which the higher hydrophobic character displayed by [Si][N_3888_]Cl leads to preferable hydrophobic interactions with tRNA, whereas IL ligands with shorter alkyl side chains have a higher charge density and preferably establish electrostatic interactions.

**Figure 3:**
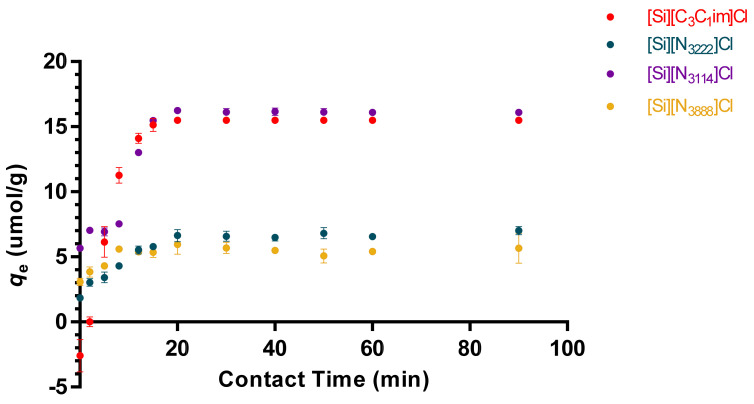
Adsorption kinetic curves of tRNA (5 µg/mL) in IL-functionalized silica supports at 25 °C. Binding buffer: 10 mM Tris-HCl, pH 8.0, for [Si][C_3_C_1_im]Cl, [Si][N_3222_]Cl, [Si][N_3114_]Cl, and 2 M (NH_4_)_2_SO_4_ in 10 mM Tris-HCl, pH 8.0, for [Si][N_3888_]Cl. All data are reported as mean ± SD, *n* = 3.

Under the described conditions, a plateau in the equilibrium concentration of adsorbate in the solid phase (*q_e_*) was reached approximately at 20 min, which was maintained up to the 90 min of time evaluated. According to the results shown in Figure 3, the initial adsorption rate of tRNA onto the material surface is high and fast, meaning an initial strong, non-covalent binding of tRNA onto the sorbent surface, followed by the saturation of the supported material at *q_e_*. Altogether, these results indicate that interactions between the modified supports and tRNA molecules are strong and occur quickly. In addition, when using 10 mM Tris-HCl as the binding buffer, the tRNA binding capacities in the solid phase reached 16.3 and 15.6 µmol.g^−1^ for [Si][N_3114_]Cl and [Si][C_3_C_1_im]Cl (the best identified materials), respectively. The maximum equilibrium concentration of adsorbate in the solid phase of the IL-modified supports decreases in the following order: [Si][N_3114_]Cl > [Si][C_3_C_1_im]Cl > [Si][N_3222_]Cl > [Si][N_3888_]Cl.

To explore the adsorption mechanisms of tRNA in the prepared IL-functionalized silica supports, pseudo first-order (PFO), pseudo second-order (PSO) and Elovich kinetic models were applied to correlate the experimental data. The data of the adsorption kinetic parameters are summarized in Table 2, whereas adsorption curves of the models are provided in Figure S3, in the Supplementary Material. According to the results, the range of the correlation coefficients (R^2^) obtained for Elovich kinetic model was between 0.610 and 0.851. In turn, the correlation coefficients for second-order (PSO) kinetic model (range between 0.919 and 0.949) are smaller than the correlation coefficients achieved for the first-order (PFO) model (higher than 0.970). Therefore, the experimental data on tRNA binding onto the modified supports correlate well with the PFO model, indicating that the adsorption process is controlled by the adsorption at the liquid–solid interface in the adsorbent [33]. This is confirmed by the higher R^2^ obtained with the PFO model (0.970), with an adsorption capacity (*K*) of ~0.142 min^−1^ and an adsorption intensity (*q*_e_) of ~6.060 mg g^−1^. In the case of the PSO model (R^2^ ~0.928), a *K* and *q*_e_ of ~0.267 g mg^−1^ min^−1^ and ~6.837 mg g^−1^ were obtained, respectively. For the Elovich model, the plateau region is not achieved (see Figure S3 in the Supplementary Material), being the R^2^ ~0.851, and β and α of ~0.772 g mg^−1^ and ~3.068 mg g^−1^ min^−1^, respectively.

### 3.3. Binding/Elution Experiments of tRNA Using IL-Functionalized Silica Supports in Batch Mode

**Figure 4:**
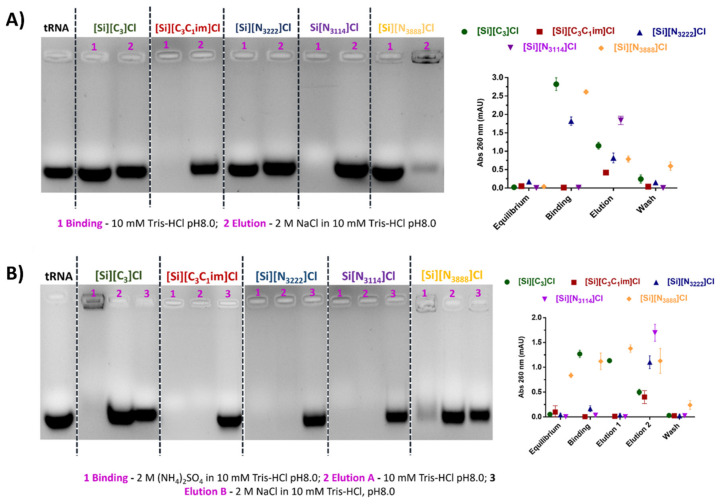
Agarose gel electrophoresis of tRNA from *E. coli* using IL-functionalized silica supports. (**A**) In ionic conditions: Lane 1: tRNA binding with 10 mM Tris-HCl, pH 8.0; Lane 2: tRNA elution with 2 M NaCl in 10 mM Tris-HCl, pH 8.0; (**B**) In hydrophobic conditions: Lane 1: tRNA binding with 2 M (NH_4_)_2_SO_4_ in 10 mM Tris-HCl, pH 8.0; Lane 2: tRNA elution with 10 mM Tris-HCl, pH 8.0. Lane 3: tRNA elution with 2 M NaCl in 10 mM Tris-HCl, pH 8.0. All data are reported as mean ± SD, *n* = 3.

The binding/elution behavior of nucleic acids onto the IL-modified supports was performed using a sample composed of tRNA, which was extracted from *E. coli* using the guanidinium thiocyanate-phenol-chloroform method. Agarose gel electrophoresis was used to detect and identify tRNAs species eluted in each chromatographic step, while the absorbance of each fraction was measured at 260 nm to infer the recovered RNA levels. Aiming to study the preferential establishment of ionic interactions, tRNA binding was promoted with 10 mM Tris-HCl, pH 8.0. This pH was selected considering that nucleic acids are negatively charged due to the phosphate groups and that SILs with significant ligand densities display PZC values ranging from 9.2 to 9.5, having thus an overall surface positive charge (Table 1). After retention, tRNA elution was then achieved by increasing the ionic strength of the buffer to 2 M NaCl in 10 mM Tris-HCl (pH 8.0). According to Figure 4A, and for each IL-modified support, the electrophoretic profiles presented in lanes 1 and 2 correspond to the concentrated and desalted samples obtained after binding with 10 mM Tris-HCl and elution with 2 M NaCl in 10 mM Tris-HCl, respectively. In these conditions, mainly favoring ionic interactions, it was observed that tRNA does not interact with [Si][N_3888_]Cl due to the higher hydrophobic character of the material. In the same line, the tRNA was only partially retained on the [Si][N_3222_]Cl support, whereas the supports [Si][N_3114_]Cl and [Si][C_3_C_1_im]Cl were able to bind all tRNA due to their higher charge density. These results suggest that RNA establish strong electrostatic interactions with these materials under the evaluated conditions. These results are in agreement with Figure 3, given that the SILs [Si][N_3114_]Cl and [Si][C_3_C_1_im]Cl present the highest equilibrium concentrations of adsorbate in the solid phase (*q_e_*).

The performance of the support [Si][C_3_]Cl was also evaluated as a control, once it only contains the spacer arm (common to all IL-modified supports), it being demonstrated that it presents a low tRNA binding ability and thereby confirms that the enhanced performance observed with the supports [Si][N_3114_]Cl and [Si][C_3_C_1_im]Cl is due to the presence of the IL as ligand.

A distinct set of experiments designed to mainly exploit hydrophobic interactions was carried out using a binding buffer containing 2 M (NH_4_)_2_SO_4_ in 10 mM Tris-HCl (pH 8.0). This salt concentration promoted total tRNA retention in all supports. Regarding the tRNA desorption, and contrarily to the [Si][N_3888_]Cl and [Si][C_3_]Cl materials where tRNA species immediately eluted with 10 mM Tris-HCl, in [Si][N_3222_]Cl, [Si][N_3114_]Cl, and [Si][C_3_C_1_im]Cl supports, tRNA elution was only achieved in a second elution step with 2 M NaCl in 10 mM Tris-HCl (see Figure 4B). Under these conditions, no major differences between the highly hydrophobic material [Si][N_3888_]Cl and [Si][C_3_]Cl were observed, suggesting that hydrophobic interactions may be responsible for the interaction between tRNA and the supports. However, by using moderately hydrophobic ILs as chromatographic ligands such as those found in [Si][N_3222_]Cl and [Si][N_3114_]Cl, a typical multimodal behavior was verified, which can be attributed to the presence of the IL.

Overall, the elution with NaCl allowed the total recovery of tRNA using the supports [Si][N_3114_]Cl and [Si][C_3_C_1_im]Cl since the biomolecule was not detected in the washing step (Figure 4B). With these supports, it is additionally observed that the binding/elution percentage is 100 (Figure 4A,B, in the agarose gel electrophoresis and UV spectroscopy), indicating no losses of the target biomolecule. Based on the exposed, the promising results regarding the high capacity and the high biomolecule recoveries (virtually 100%) displayed by the supports [Si][N_3114_]Cl and [Si][C_3_C_1_im]Cl are highly encouraging envisaging the scale up of this technology. Different types of non-covalent interactions are established with the target biomolecule, which can be explored in preparative liquid chromatography of nucleic acids towards an increase in the selectivity of such purification processes. The recognition of tRNA by the immobilized ligands can be explained by the negative charge, structural diversity and, consequently, the nucleotide bases exposure on tRNA species, characteristics that are important for their interaction with supports [36].

### 3.4. Isolation of tRNA from an Escherichia coli DH5α Extract Using IL-Functionalized Silica Supports

**Figure 5:**
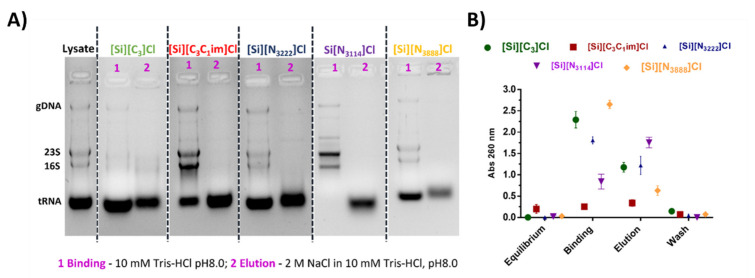
(**A**) Agarose gel electrophoresis of purified fractions from *E. coli* lysate using IL-functionalized silica supports. Lane 1: Sample recovered from the binding step; Lane 2: Sample recovered by elution with 2 M NaCl (in 10 mM Tris-HCl pH 8); (**B**) absorbance at 260 nm of the fractions recovered at different stages of the purification process. All data are reported as mean ± SD, *n* = 3.

Following the previously reported promising results, the potential application of IL-functionalized silica supports to purify tRNA directly from a clarified *E. coli* lysate containing rRNA, tRNA, and gDNA was investigated. According to the results described above, either ionic or hydrophobic interactions can be preferentially exploited using the IL-modified supports towards the purification of the target RNA. However, as the high salt concentrations required to explore hydrophobic conditions may negatively affect the stability of RNA, binding was performed with 10 mM Tris-HCl, pH 8, and the elution of retained species accomplished with an increasing NaCl (in 10 mM Tris-HCl, pH 8) stepwise gradient. The concentrated fractions were analyzed by agarose gel electrophoresis, given in Figure 5. The typical agarose gel electrophoresis profile of the bacterial lysate sample includes four well-defined bands, which sequentially correspond, in a decreasing order of molecular weight, to gDNA, rRNA 23S, rRNA 16S, and tRNA. In general, rRNA (16 and 23 S) and gDNA do not interact with the IL-modified supports (lane 1 in Figure 5A) being mainly eluted during the binding step, while tRNA seems to be preferentially retained, only eluting with the increase of the ionic strength (lanes 2 in Figure 5A). In addition, the amount of nucleic acids distributed by each fraction (binding and elution steps), evaluated by the absorbance at 260 nm (Figure 5B), is well correlated with the findings from agarose gel electrophoresis (Figure 5A). gDNA and rRNA present lower retention when compared with tRNA species, revealing a weaker interaction with the IL-modified supports. Previously, using a macroporous resin modified with [C_3_C_1_im]Cl IL as a stationary phase for preparative column liquid chromatography, Neves et al. [29] achieved the sequential separation of tRNA, gDNA, and rRNA from a complex bacterial lysate sample, using a three-step NaCl gradient consisting of 0.38 M, 0.41 M, and 0.5 M NaCl. This profile of nucleic acids elution [29] differs from that obtained in this work (cf. Figure 5), reinforcing that, in addition to the chromatographic conditions, the starting material used for the immobilization of ILs influences the separation of nucleic acids.

Nevertheless, the results herein reported are in accordance with other studies already published, where a different interaction behavior of double- and single- stranded nucleic acids during the separation process has been described [37]. The stronger and selective interaction occurring between the tRNA species and the supports can be explained by the single-stranded nature of RNA, which is usually involved in RNA recognition, owing to the high nucleotide bases exposure and availability for interactions. Hence, tRNA structural features (structure of tRNA can be decomposed into its primary structure, secondary structure—usually visualized as the cloverleaf structure-, and a tertiary structure) seem to be relevant on its distinct retention behavior on SILs. Considering the different supports under evaluation, a higher selectivity towards tRNA is achieved with [Si][N_3114_]Cl and [Si][C_3_C_1_im]Cl, which show an enhanced ability to effectively distinguish tRNA from other classes of nucleic acids (even with these species presenting similar physicochemical properties).

Overall, the immobilization of appropriately designed ILs in stationary phases and their application as supports for preparative liquid chromatography is herein demonstrated as a highly effective strategy for the purification of nucleic acids, with wider applications towards other biomolecules or nucleic acids from other sources being expected. This work proves the relevance of establishing well-defined binding/elution conditions (increased NaCl or decreased (NH_4_)_2_SO_4_ gradients) to improve the purification performance of tRNA, namely in terms of final yields of tRNA while guaranteeing an enhanced stability of the purified tRNA. The performance of ILs as chromatographic ligands for the purification of tRNA is highly dependent upon their chemical structure, being improved by ILs composed of aromatic cations and in which the positive charge center seems equally important.

Among the elution approaches evaluated—increasing NaCl or decreasing (NH_4_)_2_SO_4_ concentration gradients—the NaCl-based strategy represents an advantageous alternative to (NH_4_)_2_SO_4_, mostly due to: the low salt concentration employed, fast separation and consequent short contact time, contribution to maintain the structural stability of the target molecule, and lower eutrophication potential of NaCl in comparison to (NH_4_)_2_SO_4_. Overall, the effective performance displayed by the technology herein developed based on SILs reinforces its high potential towards the development of innovative chromatographic strategies for the purification of nucleic acids, feasible of industrial application.

## 4. Conclusions

Herein, we report the chemical modification of silica-based supports with ILs and their application as innovative chromatographic supports for the isolation and purification of RNA from complex bacterial lysates. To accomplish this goal, a two-step preparation procedure was employed, encompassing an initial reaction between activated silica and 3-chloropropyltrimethoxysilane, and then this material followed a second reaction to have a covalently attached IL cation and the chloride counter ion. The materials functionalization with ILs was confirmed by several techniques, such as solid-state ^13^C RMN, elemental analysis, and zeta potential measurements.

The adsorption kinetics of bacterial tRNA onto the modified supports disclosed that significantly higher tRNA binding capacities are obtained for [Si][N_3114_]Cl and [Si][C_3_C_1_im]Cl (16.3 and 15.6 µmol.g^−1^, respectively) in comparison with [Si][N_3222_]Cl and [Si][N_3888_]Cl. The chromatographic performance of the modified supports using an isolated bacterial tRNA sample showed that, with the exception of [Si][N_3888_]Cl, all supports bind tRNA either in conditions that mainly favor hydrophobic interactions or ionic interactions. Moreover, these supports showed themselves to be versatile, and that can be explored in a multimodal way, as they can establish and combine different types of non-covalent interactions with the target biomolecules, thereby reinforcing their high selectivity potential.

Among the studied SILs, [Si][N_3114_]Cl and [Si][C_3_C_1_im]Cl present the required selectivity for the isolation of tRNA from a complex bacterial lysate containing gDNA and rRNA, in addition to tRNA. Overall, this study discloses the potential of using ILs as chromatographic ligands immobilized in silica-based supports as innovative purification technologies for the downstream processing of RNA.

## Figures and Tables

**Table 1:** Elemental analysis (carbon, hydrogen, and nitrogen weight fraction percentage) results, ligand density and point of zero charge (PZC) for the supports SiO_2_, [Si][C_3_]Cl, [Si][C_3_C_1_im]Cl, [Si][N_3222_]Cl, [Si][N_3114_]Cl and [Si][N_3888_]Cl.

Sample	wt% C	wt% H	wt% N	Ligand Density (nmol IL per g of Silica) ^1^	PZC
[SiO_2_]	-	-	-	-	3.4
[Si][C_3_]Cl	4.73	1.44	0.00	-	4.1
[Si][C_3_C_1_im]Cl	8.21	1.55	2.50	0.89	9.5
[Si][N_3114_]Cl	7.75	1.77	0.77	0.55	9.2
[Si][N_3222_]Cl	7.29	1.51	0.26	0.19	9.2
[Si][N_3888_]Cl	6.34	1.66	0.06	0.05	6.2

^1^ Determined by Q = [(wt% nitrogen)/(1.4 × number of nitrogen atoms)].

**Table 2:** Experimental data (*q*_e_) and modeling results obtained with pseudo first-order, pseudo second-order, and Elovich kinetic models for [Si][C_3_C_1_im]Cl, [Si][N_3222_]Cl, [Si][N_3114_]Cl and [Si][N_3888_]Cl.

Model/SIL	PFO	PSO	Elovich
[Si][C_3_C_1_im]Cl	*q*_e_ = 6.060 mg g^−1^ *K*_1_ = 0.142 min^−1^ R^2^ = 0.970	*q*_e_ = 6.837 mg g^−1^ *K*_2_ = 0.267 g mg^−1^ min^−1^ R^2^ = 0.928	β = 0.773 g mg^−1^ α = 3.068 mg g^−1^ min^−1^ R^2^ = 0.851
[Si][N_3114_]Cl	*q*_e_ = 6.607 mg g^−1^ *K*_1_ = 0.131 min^−1^ R^2^ = 0.981	*q*_e_ = 7.326 mg g^−1^ *K*_2_ = 0.265 g mg^−1^ min^−1^ R^2^ = 0.949	β = 0.885 g mg^−1^ α = 7.787 mg g^−1^ min^−1^ R^2^ = 0.835
[Si][N_3222_]Cl	*q*_e_ = 6.060 mg g^−1^ *K*_1_ = 0.142 min^−1^ R^2^ = 0.970	*q*_e_ = 6.837 mg g^−1^ *K*_2_ = 0.267 g mg^−1^ min^−1^ R^2^ = 0.928	β = 0.773 g mg^−1^ α = 3.068 mg g^−1^ min^−1^ R^2^ = 0.851
[Si][N_3888_]Cl	*q*_e_ = 5.424 mg g^−1^ *K*_1_ = 0.615 min^−1^ R^2^ = 0.988	*q*_e_ = 5.643 mg g^−1^ *K*_2_ = 0.212 g mg^−1^ min^−1^ R^2^ = 0.919	β = 2.555 g mg^−1^ α = 1.428 mg g^−1^ min^−1^ R^2^ = 0.610

## Data Availability

Data is contained within the article or Supplementary Material.

## Supplementary Materials

The following are available online at https://www.mdpi.com/article/10.3390/life11101090/s1, Figure S1: Zeta potential values as a function of pH and values of point of zero charge (PZC) for SiO_2_, [Si][C_3_]Cl and IL-functionalized silica supports, Figure S2: SEM images of [Si][C_3_]Cl and IL-functionalized silica supports, Figure S3: Fitting of the adsorption kinetic data of tRNA onto IL-functionalized silica supports at 25 °C by the PFO, PSO, and Elovich models.
