# Supplementary material for: Efficient Isolation of Bacterial RNAs Using Silica-Based Materials Modified with Ionic Liquids
Source: Life (Basel). 2021 Oct 15;11(10):1090. doi: 10.3390/life11101090 (PMC8536996; doi:10.3390/life11101090)
Supplement: Supplementary file 1 [file life-11-01090-s001.zip › life-1389735-supplementary.pdf]

life

MDPI

Article

Efficient Isolation of Bacterial RNAs Using Silica-Based Materials Modified with Ionic Liquids

Patrícia Pereira [1,2,†], Augusto Q. Pedro [3,†], Márcia C. Neves [3], João C. Martins [4], Inês Rodrigues [4], Mara G. Freire [3,*], and Fani Sousa [4,*]

[1] CEMMPRE, Department of Chemical Engineering, University of Coimbra, Rua Sílvio Lima-Pólo II, 3030-790 Coimbra, Portugal; papereira@ipn.pt
[2] IPN, Instituto Pedro Nunes, Associação para a Inovação e Desenvolvimento em Ciência e Tecnologia, Rua Pedro Nunes, 3030-199 Coimbra, Portugal
[3] CICECO—Aveiro Institute of Materials, Department of Chemistry, University of Aveiro, 3810-193 Aveiro, Portugal; apedro@ua.pt (A.Q.P.); mcneves@ua.pt (M.C.N.)
[4] CICS-UBI—Health Sciences Research Centre, Universidade da Beira Interior, 6200-506 Covilhã, Portugal; joao_martins1994@hotmail.com (J.C.M.); ines_rodrigues@hotmail.com (I.R.)
* Correspondence: maragfreire@ua.pt (M.G.F.); fani.sousa@fcsaude.ubi.pt (F.S.); Tel.: +35-12-3440-1422 (M.G.F.); +35-12-7532-9074 (F.S.)
† These authors contributed equally to this work

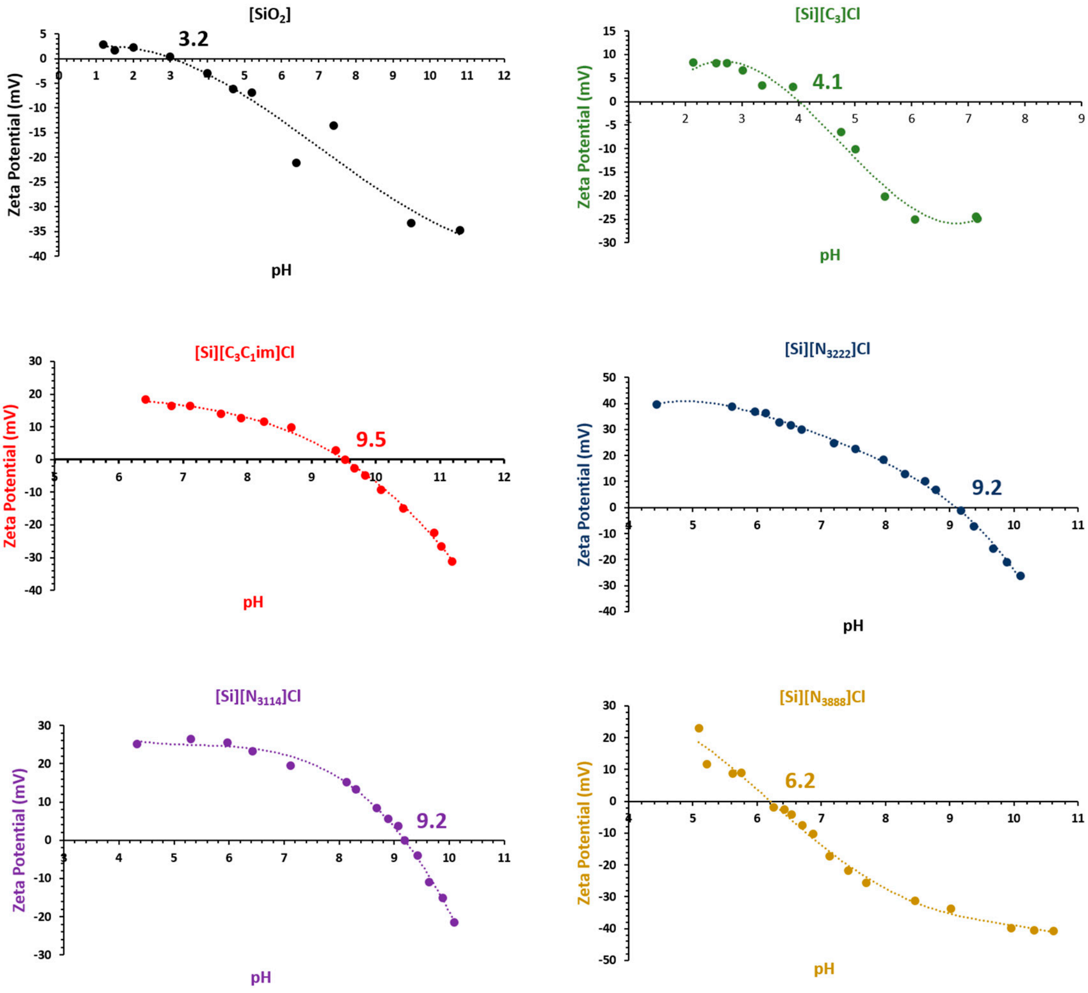

Figure S1. – Zeta potential values as a function of pH and values of point of zero charge (PZC) for SiO_2, $[Si][C_3]Cl$ and IL-functionalized silica supports.

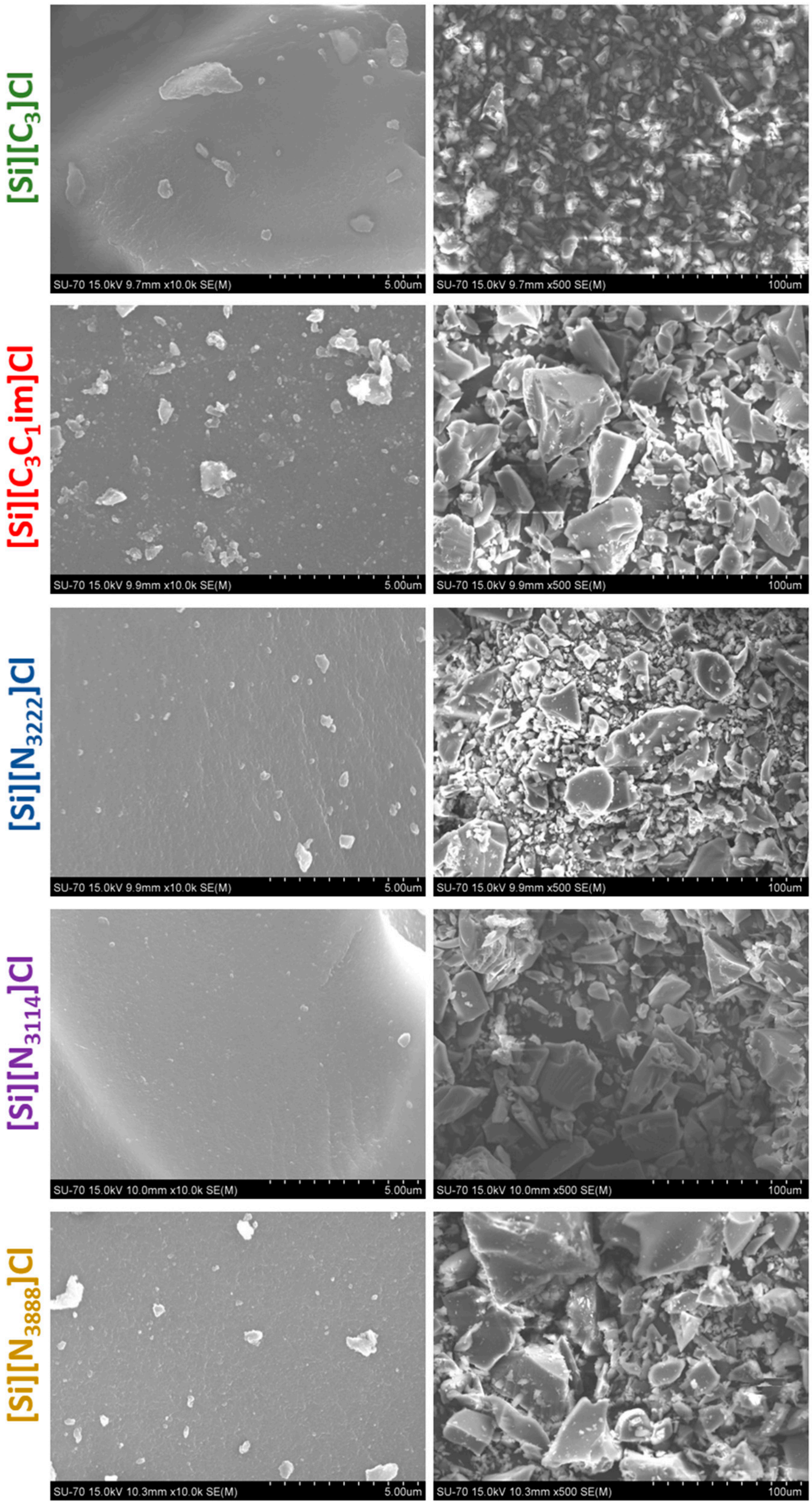

Figure S2. - SEM images of [Si][C_3]Cl and IL-functionalized silica supports.

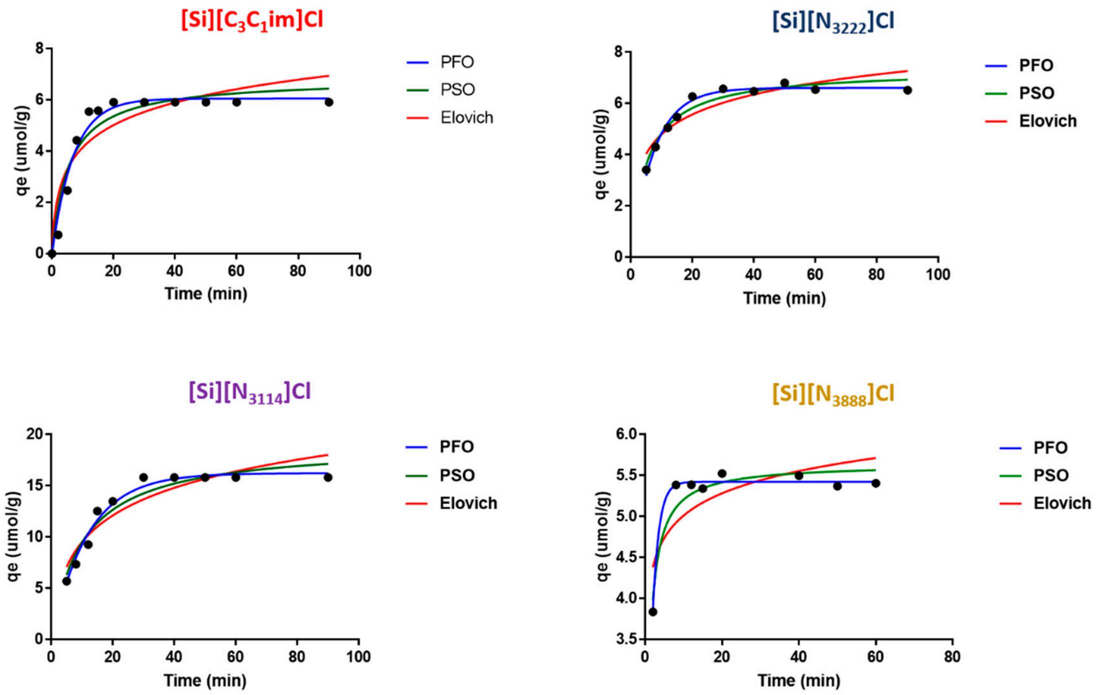

Figure S3. – Fitting of the adsorption kinetic data of tRNA onto IL-functionalized silica supports at 25°C by the PFO, PSO, and Elovich models.
